# Influenza Vaccine Uptake in the Year After Concurrent vs Separate Influenza and Zoster Immunization

**DOI:** 10.1001/jamanetworkopen.2021.35362

**Published:** 2021-11-19

**Authors:** Benjamin N. Rome, William B. Feldman, Michael A. Fischer, Rishi J. Desai, Jerry Avorn

**Affiliations:** 1 Division of Pharmacoepidemiology and Pharmacoeconomics, Department of Medicine, Brigham and Women’s Hospital, Boston, Massachusetts; 2 Harvard Medical School, Boston, Massachusetts; 3 Division of Pulmonary and Critical Care Medicine, Department of Medicine, Brigham and Women’s Hospital, Boston, Massachusetts

## Abstract

**Importance:**

Fewer than half of US adults receive the influenza vaccine each year; many cite concerns about side effects, which occur infrequently. By contrast, the recombinant zoster vaccine causes systemic side effects in a large proportion of patients.

**Objective:**

To determine whether concurrent administration of the influenza and zoster vaccines was associated with a reduced likelihood of influenza vaccination in the subsequent year.

**Design, Setting, and Participants:**

This cohort study included patients aged 50 years or older who received the influenza vaccine between August 1, 2018, and March 31, 2019, and received the zoster vaccine on the same day or separately (within the prior 180 days). Data were gathered from a national claims database of patients with commercial insurance and Medicare Advantage plans. Logistic regression analysis was used to adjust for baseline demographic characteristics, comorbidities, influenza vaccine month and location (pharmacy vs medical office), and health care use (including influenza vaccination in the prior year).

**Exposures:**

Concurrent vs separate influenza and zoster vaccine administration.

**Main Outcomes and Measures:**

Receipt of the influenza vaccine in the subsequent year (August 1, 2019, to March 31, 2020).

**Results:**

Among 89 237 individuals included in this study, the median age was 72 years (IQR, 67-77 years), 58.3% were women, 70.1% were White, and 85.7% had at least 1 comorbidity. Influenza vaccine uptake in 2019-2020 was lower among 27 161 individuals who received concurrent influenza and zoster vaccines compared with the 62 076 individuals who received the vaccines on separate days (87.3% vs 91.3%; adjusted odds ratio, 0.74; 95% CI, 0.71-0.78; *P* < .001). Results were similar across subgroups.

**Conclusions and Relevance:**

Results of this cohort study suggest that concurrent administration of influenza and zoster vaccines was associated with a reduction in receipt of the influenza vaccine the following year. One possible explanation is that some patients could have misattributed systemic side effects caused by the zoster vaccine to the influenza vaccine. It may be preferable to administer these 2 vaccines separately or enhance patient counseling about expected vaccine side effects.

## Key Points

**Question:**

Are patients who receive the influenza and recombinant zoster vaccines on the same day less likely to receive an influenza vaccine the following year?

**Findings:**

In this national cohort study of 89 237 patients, those who received concurrent influenza and zoster vaccines were significantly less likely to receive an influenza vaccine the following year (87.3% vs 91.3%).

**Meaning:**

Concurrent administration of the influenza and zoster vaccines was associated with lower uptake of the influenza vaccine the following year, possibly because patients misattributed side effects commonly caused by the zoster vaccine to the influenza vaccine.

## Teaser

This cohort study uses data from a national claims database to investigate the association of concurrent administration of the influenza and zoster vaccines with influenza vaccine uptake the following year in patients older than 50 years.

## Introduction

Despite its being universally recommended, fewer than half of US adults receive an influenza vaccine each year.^1,2^ Among those who choose not to get vaccinated, 3 in 10 cite concerns about potential side effects.^3^ This vaccine hesitancy based on perceived side effects exists despite evidence that rates of systemic side effects (eg, fatigue, myalgias, headaches, and fever or chills) are comparable among patients receiving the influenza vaccine and those receiving a placebo.^4,5^ Only local injection site symptoms (eg, pain, redness, or swelling) are known to be caused by the influenza vaccine.

By contrast, the recombinant varicella zoster vaccine used to prevent shingles is often highly reactogenic, causing systemic reactions in half of patients compared with one-quarter of patients receiving a placebo.^6^ The recombinant zoster vaccine, approved in 2017, has now replaced the live attenuated zoster vaccine, which caused fewer side effects but was far less effective in preventing shingles among older adults.^7^ The recombinant vaccine is now recommended for most adults aged 50 years or older as a 1-time, 2-dose series.^1^

Coadministration of vaccines is common in medical offices and pharmacies because patients are often due for multiple vaccines at the same time, and administration of more than 1 vaccine is not generally thought to undermine efficacy.^8,9^ A single randomized clinical trial^10^ specifically evaluated coadministration of the influenza and recombinant zoster vaccines and found similar immunogenicity compared with separate administration of the 2 vaccines. However, self-reported local and systemic side effects were more common after the zoster vaccine than the influenza vaccine and were even more frequent when the 2 vaccines were administered together.^10^

If the zoster vaccine is due at the same time as other recommended vaccines, including the annual influenza vaccine, the US Centers for Disease Control and Prevention recommends simultaneous administration.^11^ However, given the different reactogenicity of the zoster and influenza vaccines and the fact that so many Americans avoid the influenza vaccine because of concerns about side effects, receiving the 2 vaccines together could lower patients’ willingness to receive influenza vaccines in subsequent years because of misattribution of the systemic side effects caused by the zoster vaccine to the influenza vaccine. We investigated whether concurrent administration of the influenza and zoster vaccines was associated with lower adherence to a subsequent influenza vaccine compared with separate administration of the 2 vaccines.

## Methods

### Data Source

For this cohort study, we used a national, commercial health insurance claims database that includes approximately 17 million patients with commercial insurance and Medicare Advantage plans in all 50 states at any given time (Optum’s deidentified Clinformatics Data Mart Database). We obtained approval from the Mass General Brigham Institutional Review Board to waive patient consent and use deidentified claims data for this study. This study followed the Strengthening the Reporting of Observational Studies in Epidemiology (STROBE) reporting guideline.

### Patient Selection and Exposure

We identified all patients who received an influenza vaccine between August 1, 2018, and March 31, 2019, and received a dose of the recombinant zoster vaccine either on the same day (concurrent administration) or 29 to 180 days before the influenza vaccine (separate administration). We excluded patients who received the zoster vaccine 1 to 28 days before the influenza vaccine to allow for a washout period of any potential side effects of the zoster vaccine alone. Patients who received their second zoster vaccine on the same day as the influenza vaccine (ie, had received a previous zoster vaccine dose at least 28 days earlier) were included in the concurrent administration group; we performed a subgroup analysis in which we stratified concurrent administration of the influenza vaccine with the first vs second dose of the zoster vaccine. In a sensitivity analysis, we expanded the separate administration group to include patients who received a dose of the zoster vaccine 29 to 180 days after their index influenza vaccine; this was done to address potential selection bias among the separate administration group because these patients chose to receive their index influenza vaccine after a zoster vaccine. We measured vaccines administered both in office settings (using *Current Procedural Terminology* codes in medical claims) and at pharmacies (using National Drug Codes in pharmacy claims).

**Figure 1.**
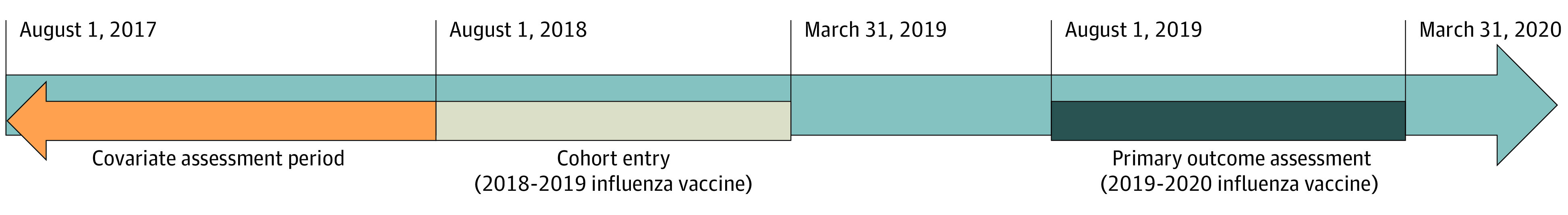
Study Design Patients entered the cohort when they received their 2018-2019 influenza vaccine. The covariate assessment period was August 1, 2017, through the date of cohort entry, with most covariates measured in the 365 days before cohort entry. Follow-up for the primary outcome was during the 2019-2020 influenza vaccine season. Secondary (negative control) outcomes were measured from the day after cohort entry through March 2020.

Patients were required to have continuous insurance enrollment (allowing for a 30-day grace period of gaps in coverage) from August 1, 2017, through the date of their 2018-2019 influenza vaccine. This timeline served as the covariate assessment period to measure baseline characteristics and prior health care use, with most covariates measured in the 365 days before cohort entry (Figure 1). We excluded patients aged younger than 50 years (because the recombinant zoster vaccine is not indicated), those with prior concurrent administration of the influenza and zoster vaccines (so that the concurrent administration group represented first exposure), and those with multiple influenza vaccines during the 2018-2019 season. We also excluded a small number of people with missing sex data, who lived outside the US, or who received the live intranasal influenza vaccine, which is not recommended for patients aged older than 50 years.

### Adjustment for Confounders

We adjusted for factors that could differ between patients who received concurrent vs separate administrations of the influenza and zoster vaccines, including demographic, clinical, and health care use variables assessed during the covariate assessment period. The demographic characteristics that we adjusted for included age (in deciles), sex, race and ethnicity (defined in the data set as Asian, Black, Hispanic, White, or Missing and derived from a combination of sources), geographic region, insurance type (commercial vs Medicare Advantage), month and location of the 2018-2019 influenza vaccine (office vs pharmacy), type of influenza vaccine (standard, high dose, recombinant, adjuvanted, or unknown), and concurrent administration of any additional vaccines besides the zoster vaccine (eg, pneumococcal or tetanus vaccines). For the small number of patients who had both a medical and pharmacy claim for the influenza vaccine on the same day, we assumed that the vaccine was picked up from a pharmacy and administered in the physician’s office. We included race and ethnicity as possible confounders due to known disparities in annual influenza vaccination rates.^2^

We also adjusted for several comorbidities known to increase the risk of complications from influenza, including hypertension, diabetes, cardiovascular disease, chronic obstructive pulmonary disease or asthma, chronic kidney disease, or liver disease, and immunocompromising conditions.^12,13,14^ We measured each condition based on the *International Statistical Classification of Diseases and Related Health Problems, Tenth Revision* codes on any inpatient or outpatient claim during the 365 days before and including the date of the patient’s 2018-2019 influenza vaccination. For immunocompromising conditions, we included a diagnosis of HIV infection as well as exposure to potentially immunosuppressive drugs, including chemotherapy (World Health Organization Anatomical Therapeutic Chemical [WHO ATC] classification system L01), corticosteroids (WHO ATC H02, M01BA), or other immunosuppressants (WHO ATC L04).

We also measured prior health care use, including number of office visits and filled prescriptions, visits to primary care clinicians, routine laboratory measurements (basic metabolic panel, complete blood count, hemoglobin A_1c_, and lipids), emergency department visits, inpatient hospitalizations, and preventive services (screening for colon, breast, and prostate cancer; tetanus and pneumococcal vaccination; and bone mineral density testing) during the 365 days before 2018-2019 influenza vaccination. We also measured whether patients had received an influenza vaccine during the prior season (August 1, 2017-March 31, 2018), as patients who received 2 consecutive influenza vaccines would be expected to have a higher likelihood of receiving a third vaccine in 2019-2020.

To assess potential residual confounding, we also measured patients’ use of preventive care services during the follow-up period as negative control outcomes^15^ starting the day after their 2018-2019 influenza vaccination through March 2020. We selected 6 preventive services that are performed routinely in most adults aged 50 years or older, including colon cancer screening (eg, colonoscopy, fecal immunohistochemical testing), breast cancer screening, prostate cancer screening (prostate-specific antigen testing), bone mineral density testing, pneumococcal vaccination (PPSV23 or PCV13), and tetanus vaccination (Tdap or Td).^16^ We performed these analyses among the entire population, adjusting for age and sex, and we also analyzed each negative control outcome among the age- and sex-defined subgroup in which the preventive service is routinely recommended (eg, women aged 50-74 years for breast cancer screening). Finally, we performed a quantitative bias analysis using an established approach to assess whether the results from our primary analysis could be explained by residual confounding by socioeconomic status, which is associated with the outcome (influenza vaccination) and is not measured in the claims data (eFigure 3 in the Supplement).^17,18^

### Primary Outcome

We used pharmacy and medical claims to measure whether patients received a subsequent influenza vaccine in the 2019-2020 season (August 1, 2019, through March 31, 2020). In the primary analysis, we included only patients who had continuous enrollment through March 2020. In a sensitivity analysis, we included all patients regardless of whether they died or disenrolled from the insurance plan before March 2020.

### Statistical Analysis

We performed logistic regression to estimate the adjusted odds of receiving a 2019-2020 influenza vaccine after concurrent influenza and zoster vaccine administration in the prior year compared with separate administration of the 2 vaccines. We performed subgroup analyses based on baseline characteristics. We adjusted for the same set of potential confounders in the primary and sensitivity analyses, in subgroup analyses, and when evaluating negative controls. All *P* values were 2-sided, and significance was defined as <.05. The cohort was generated and outcomes measured using the Aetion Evidence Platform (Aetion Inc).^19^ Statistical analyses were performed using SAS software, version 9.4 (SAS Institute Inc).

## Results

### Cohort Characteristics

**Figure 2.**
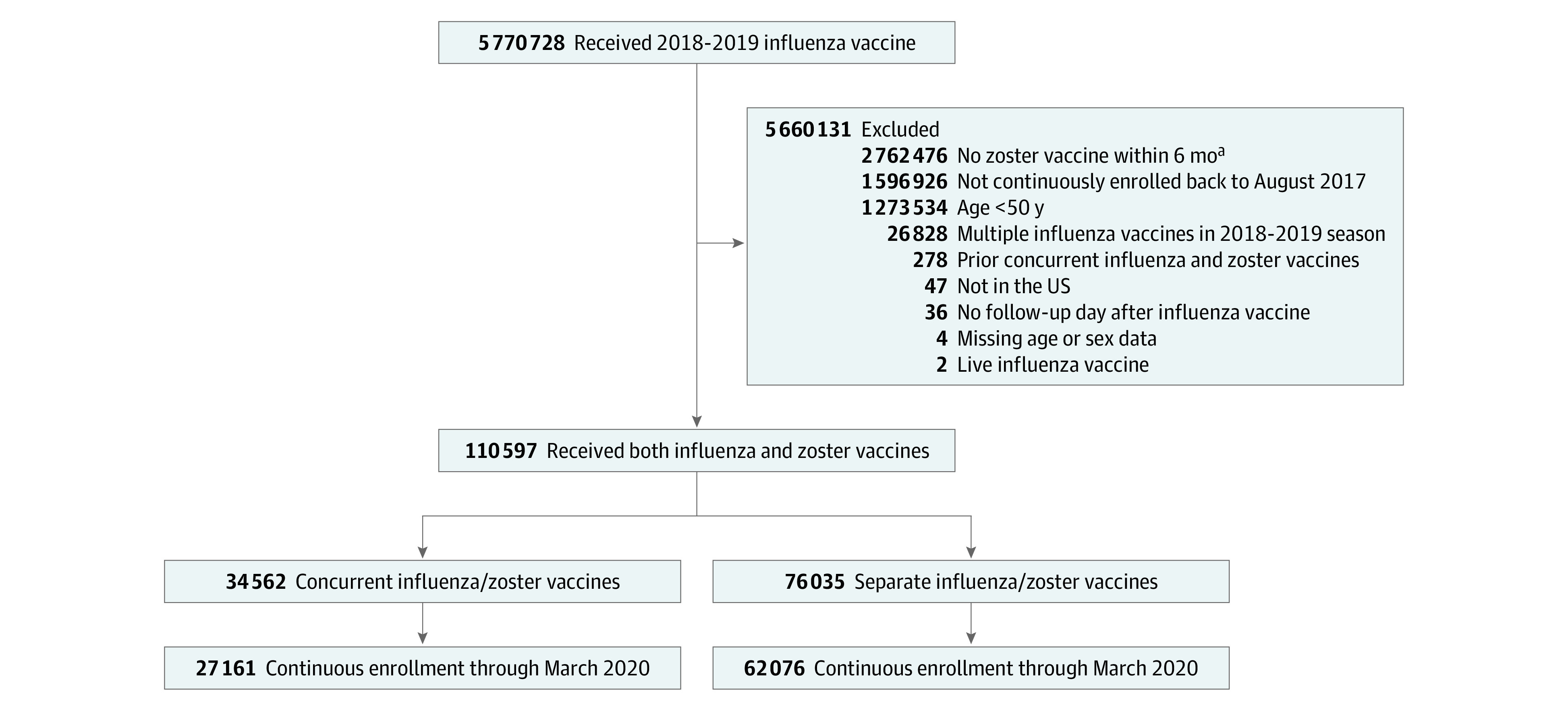
Cohort Selection and Follow-up ^a^Zoster vaccine must have been administered either on the same day as the 2018-2019 influenza vaccine or between 29 and 180 days before the influenza vaccine was administered.

In our primary analysis, we studied 89 237 patients, including 27 161 (30.4%) who received the 2018-2019 influenza and zoster vaccines on the same day and 62 076 (69.6%) who received the vaccines on separate days. The reasons for exclusion are shown in Figure 2. The median age of the cohort was 72 years (IQR, 67-77 years), 58.3% were women, 41.7% were men, 7.1% were Asian, 7.5% were Black, 4.0% were Hispanic, 70.1% were White, and 11.2% were an unknown race or ethnicity. A total of 85.7% had at least 1 comorbidity that increased their risk for complications from influenza (most commonly hypertension [67.8%], cardiovascular disease [31.3%], use of any oral corticosteroids [29.0%], and/or diabetes [26.5%]) (Table). Patients who received influenza and zoster vaccines concurrently were younger (43.4% vs 32.9% aged younger than 70 years) and less likely to be enrolled in Medicare Advantage plans (77.5% vs 86.6%) compared with those who received the 2 vaccines separately.

**Table.** Cohort Characteristics, Stratified by Exposure Group

Characteristic	Concurrent vaccines (n = 27 161), No. (%)	Separate vaccines (n = 62 076), No. (%)	Standardized difference
Age, y			
50-59	3254 (12.0)	3823 (6.2)	0.2038
60-69	8533 (31.4)	16 551 (26.7)	0.1049
70-79	11 487 (42.3)	29 982 (48.3)	0.1209
≥80	3887 (14.3)	11 720 (18.9)	0.1230
Sex			
Women	15 551 (57.3)	36 474 (58.8)	0.0304
Men	11 610 (42.7)	25 602 (41.2)	
Race and ethnicity			
Asian	2264 (8.3)	4094 (6.6)	0.0663
Black	2319 (8.5)	4380 (7.1)	0.0553
Hispanic	937 (3.4)	2639 (4.3)	0.0417
White	18 727 (68.9)	43 867 (70.7)	0.0374
Unknown	2914 (10.7)	7096 (11.4)	0.0224
Region			
Northeast	1963 (7.2)	4873 (7.9)	0.0236
South	11 193 (41.2)	25 971 (41.8)	0.0127
Midwest	6362 (23.4)	12 687 (20.4)	0.0722
West	7643 (28.1)	18 545 (29.9)	0.0382
Medicare Advantage	21 062 (77.5)	53 787 (86.6)	0.2391
Health care use			
2017-2018 Influenza vaccine	22 593 (83.2)	55 153 (88.8)	0.1639
Hospitalization	2380 (8.8)	5303 (8.5)	0.0078
Emergency department visit	5775 (21.3)	13 031 (21.0)	0.0066
Primary care visit	21 657 (79.7)	50 761 (81.8)	0.0517
Basic metabolic panel testing	23 033 (84.8)	54 589 (87.9)	0.0915
Complete blood count testing	20 192 (74.3)	47 921 (77.2)	0.0667
Lipid testing	20 539 (75.6)	48 182 (77.6)	0.0472
Hemoglobin A_1c_ testing	12 823 (47.2)	29 436 (47.4)	0.0042
No. of office visits			
0-3	7076 (26.1)	12 858 (20.7)	0.1264
4-7	8481 (31.2)	19 169 (30.9)	0.0075
8-11	5225 (19.2)	13 269 (21.4)	0.0532
≥12	6379 (23.5)	16 780 (27.0)	0.0817
No. of filled prescriptions			
0-11	5126 (18.9)	9010 (14.5)	0.1171
12-23	5732 (21.1)	14 114 (22.7)	0.0395
24-47	8190 (30.2)	20 315 (32.7)	0.0554
≥48	8113 (29.9)	18 637 (30.0)	0.0033
Preventive health care services			
Tetanus vaccination	3039 (11.2)	7095 (11.4)	0.0076
Pneumococcal vaccination	6713 (24.7)	14 878 (24.0)	0.0174
Colon cancer screening	2871 (10.6)	6876 (11.1)	0.0163
Breast cancer screening	6114 (22.5)	15 119 (24.4)	0.0436
Prostate cancer screening	6452 (23.8)	14 724 (23.7)	0.0008
Bone mineral density testing	3698 (13.6)	10 176 (16.4)	0.0778
Risk factors for severe influenza			
Any^a^	22 838 (84.1)	53 595 (86.3)	0.0635
Diabetes	7292 (26.8)	16 396 (26.4)	0.0098
Hypertension	18 006 (66.3)	42 471 (68.4)	0.0453
Cardiovascular disease	7967 (29.3)	19 971 (32.2)	0.0616
Asthma or COPD	4927 (18.1)	11 155 (18.0)	0.0044
Liver disease	3427 (12.6)	8589 (13.8)	0.0360
Chronic kidney disease	1385 (5.1)	3233 (5.2)	0.0049
Human immunodeficiency virus	135 (0.5)	240 (0.4)	0.0166
Chemotherapy	3168 (11.7)	7482 (12.1)	0.0120
Immunosuppressant	604 (2.2)	1536 (2.5)	0.0165
Corticosteroid	8004 (29.5)	17 911 (28.9)	0.0135
Influenza vaccine type			
Standard dose	8008 (29.5)	15 831 (25.5)	0.0892
High dose	14 911 (54.9)	33 930 (54.7)	0.0048
Adjuvanted	3151 (11.6)	10 560 (17.0)	0.1550
Recombinant	337 (1.2)	275 (0.4)	0.0874
Unknown	754 (2.8)	1480 (2.4)	0.0247
Influenza vaccine at a pharmacy	15 732 (57.9)	32 487 (52.3)	0.1125
Additional vaccine(s) administered concurrently with 2018-2019 influenza vaccine^b^	2608 (9.6)	2408 (3.9)	0.2298
Month of influenza vaccine			
August 2018	3390 (12.5)	2878 (4.6)	0.2832
September 2018	11 945 (44.0)	20 576 (33.1)	0.2239
October 2018	8318 (30.6)	29 750 (47.9)	0.3599
November 2018	1893 (7.0)	6366 (10.3)	0.1173
December 2018	783 (2.9)	1608 (2.6)	0.0179
January 2019	440 (1.6)	686 (1.1)	0.0444
February 2019	250 (0.9)	159 (0.3)	0.0869
March 2019	142 (0.5)	53 (0.1)	0.0795

Abbreviation: COPD, chronic obstructive pulmonary disease.

^a^ Includes patients with 1 or more of the other listed risk factors. This variable was not included in the regression models, as each individual risk factor was included separately.

^b^ Included any vaccine for subcutaneous or intramuscular injection recommended for use in adults. The most commonly administered vaccines were conjugate and polysaccharide pneumococcal vaccines, followed by tetanus vaccines. Less common vaccines included hepatitis A, hepatitis B, varicella, meningococcus, *Haemophilus influenzae* type B, typhoid, yellow fever, Japanese encephalitis, and rabies.

More than one-half of patients received their 2018-2019 flu vaccine at a pharmacy, which was slightly more common among patients who received a concurrent zoster vaccine compared with those who received the zoster vaccine separately (57.9% vs 52.3%). Among patients with concurrent vaccine administration, 86.5% received both vaccines at the same location, and 13.2% received their zoster vaccine at the pharmacy and their influenza vaccine in an office (eFigure 1 in the Supplement). The most common types of flu vaccine were the high dose (54.7%), standard dose (26.7%), and adjuvanted (15.4%) vaccines.

### Primary Outcome

Among 27 161 patients with concurrent administration of the 2018-2019 influenza vaccine and zoster vaccine, 23 717 (87.3%) received a subsequent influenza vaccine in 2019-2020 compared with 56 686 (91.3%) of the 62 076 patients who received the 2 vaccines separately, for an absolute difference of 4.0% (95% CI, 3.5%-4.5%). After adjusting for demographic, clinical, and health care use characteristics, the adjusted odds ratio (OR) of receiving a 2019-2020 influenza vaccine was 0.74 (95% CI, 0.71-0.78; *P* < .001) following concurrent vs separate administration of the influenza and zoster vaccines in 2018-2019.

### Subgroup Analyses

**Figure 3.**
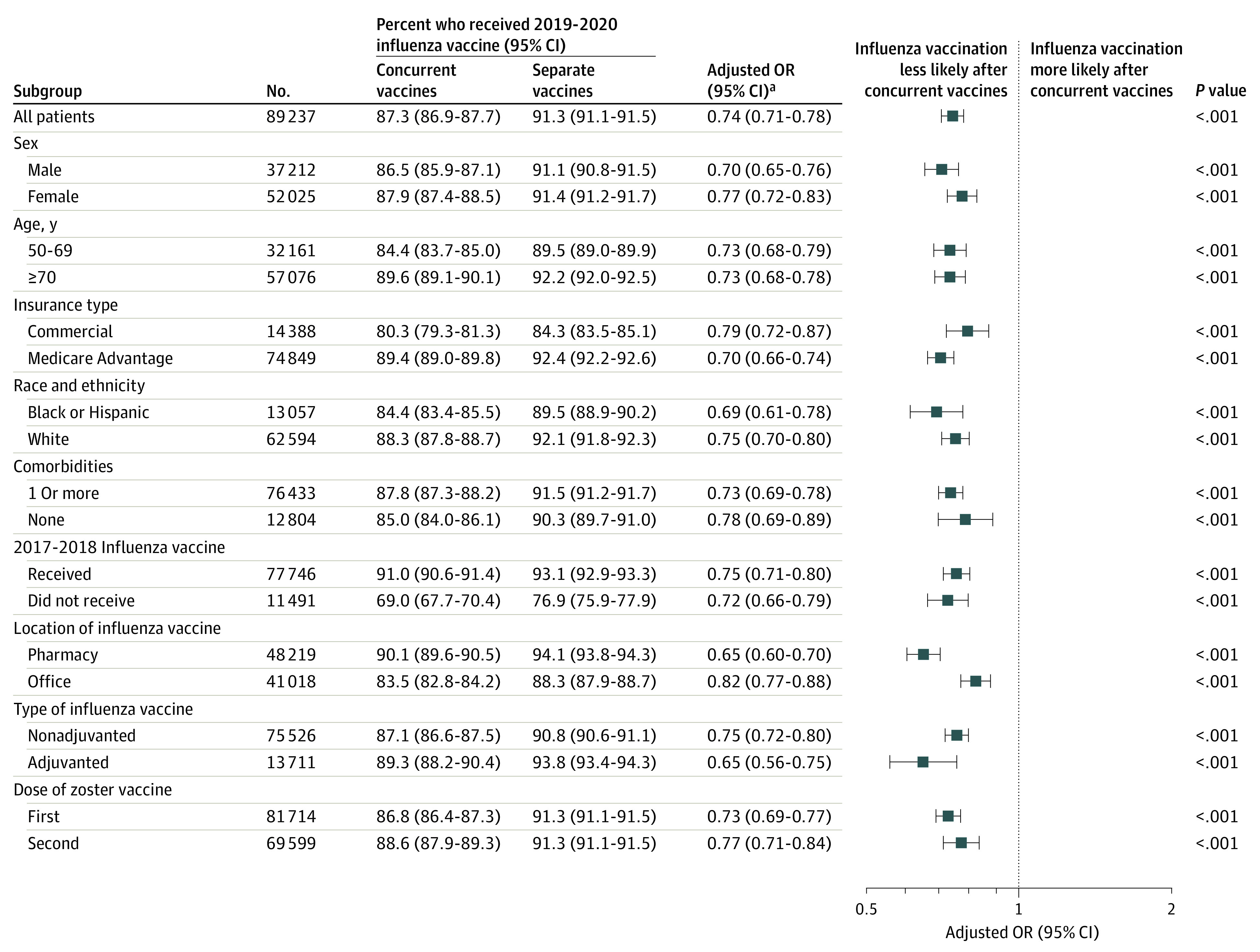
Receipt of 2019-2020 Influenza Vaccine Among Subgroups ^a^The adjusted odds of receiving an influenza vaccine in 2019-2020 after concurrent vs separate influenza and zoster vaccines in 2018-2019. Values <1.0 represent a lower odds of the outcome among patients with concurrent vaccines. OR indicates odds ratio.

Among all demographic and clinical subgroups, patients who received concurrent influenza and zoster vaccines had lower odds of receiving a 2019-2020 influenza vaccine compared with those who received the vaccines separately (Figure 3). The only covariate that was associated with different effect estimates was the location at which patients received their 2018-2019 influenza vaccine. Among patients who received their influenza vaccine at a pharmacy, concurrent zoster vaccination was associated with a 0.65 (95% CI, 0.60-0.70) adjusted odds of receiving a subsequent influenza vaccine compared with separate administration; for those who received their influenza vaccine at a medical office, the adjusted OR was 0.82 (95% CI, 0.77-0.88).

Patients more likely to receive a 2019-2020 influenza vaccine included those who received a 2017-2018 influenza vaccine compared with those who did not (92.5% vs 73.8%), those who were covered by Medicare Advantage compared with commercial insurance plans (91.5% vs 82.6%), and those who received the 2018-2019 flu vaccine at a pharmacy compared to a medical office (92.8% vs 87.0%) (eTable 1 in the Supplement).

### Negative Control Outcomes

**Figure 4.**
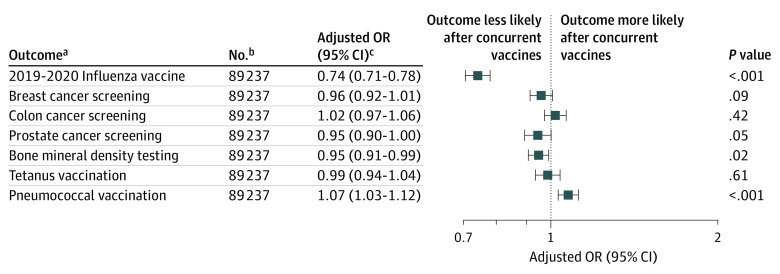
Adjusted Odds of the Primary and Negative Control Outcomes Following Concurrent vs Separate Influenza and Zoster Vaccines ^a^The primary outcome (2019-2020 influenza vaccine) was measured from August 2019-March 2020. All secondary outcomes were measured from 1 day after a patient’s 2018-2019 influenza vaccine through March 2020. Each outcome was measured among the entire cohort of patients who received concurrent influenza and zoster vaccines (n = 27 161) and those receiving separate vaccines (n = 62 076). ^b^Each negative control analysis was run using the same cohort as the primary analysis; the adjustment model included age and sex. An alternate set of analyses is shown in eFigure 2 in the Supplement, whereby each outcome was evaluated in age- and sex-defined subgroups for which each preventive service is routinely recommended. ^c^The adjusted odds of experiencing the given outcome in 2019-2020 after concurrent vs separate influenza and zoster vaccines in 2018-2019. Each outcome model was adjusted for all of the demographic, clinical, and health care use measures shown in the Table. Odds ratios (ORs) smaller than 1.0 represent a lower odds of the outcome among patients with concurrent vaccines.

To address the possibility that the observed difference was the result of residual confounding from unmeasured covariates in the 2 study populations, we also measured the proportion of patients undergoing other routine preventive care services in the year after their 2018-2019 influenza vaccine. Rates of these services ranged from 10% for tetanus vaccination to 27% for breast cancer screening. The absolute use of these services was similar for patients who received concurrent vs separate influenza and zoster vaccines (absolute risk differences ranging from −1.4% to 1.9%) (eTable 2 in the Supplement). The adjusted odds of receiving these preventive services after concurrent vs separate influenza and zoster vaccine administration ranged from 0.95 (95% CI, 0.91-0.99) for bone mineral density testing to 1.07 (95% CI, 1.03-1.12) for pneumococcal vaccination (Figure 4). The 4 other preventive services showed no significant differences in use after concurrent vs separate influenza and zoster vaccines. Results were similar when we limited each negative control outcome to the age- and sex-defined subpopulation in which the preventive service is routinely recommended (eFigure 2 in the Supplement).

### Sensitivity Analyses

In a sensitivity analysis that included an additional 61 219 patients who received the zoster vaccine 29 to 180 days after their 2018-2019 influenza vaccine rather than before it, patients who received concurrent vaccines remained less likely to receive a subsequent influenza vaccine than patients who received the 2 vaccines separately (87.3% vs 91.3%; adjusted OR, 0.70; 95% CI, 0.67-0.73; *P* < .001). In a separate analysis in which we included an additional 21 360 patients who did not have continuous insurance enrollment through March 2020, results were similar (75.3% vs 81.6%; adjusted OR, 0.84; 95% CI, 0.81-0.86, *P* < .001). Finally, in our quantitative bias analysis for unmeasured confounding, we found that the results from the primary analysis remained below the null (OR ≤0.9) even assuming an association between low socioeconomic status and influenza vaccination (OR = 0.6) and a much higher prevalence of low socioeconomic status in the concurrent vaccine group (50% vs 10%) (eFigure 3 in the Supplement).

## Discussion

In this cohort study, compared with patients who received the influenza and zoster vaccines on separate days, those who received the vaccines on the same day were significantly less likely to receive the influenza vaccine the following year. This finding held true after controlling for numerous confounders and across patients with a wide range of demographic and clinical characteristics.

The safety and efficacy of the influenza vaccine is supported by numerous clinical trials and decades of real-world experience.^4,5,20^ Yet, influenza vaccine hesitancy remains prevalent in the US, driven by a complex combination of individual (eg, knowledge, risk perception), contextual (eg, health care access, politics), and sociodemographic factors.^18,21^ Concerns about perceived side effects are a commonly reported reason that patients choose not to receive the influenza vaccine.^22^ Although evidence from 1 randomized clinical trial^10^ suggests that the influenza and zoster vaccines are equally effective when given simultaneously, patients who were coadministered vaccines in that trial were more than twice as likely to report local and systemic side effects compared with patients who received the influenza vaccine alone. We were unable to directly measure side effects in this study because these are typically mild and do not result in encounters that would be recorded in health care administration data. Nonetheless, our findings were consistent with our a priori hypothesis that greater side effects after concurrent administration of the vaccines may discourage patients from receiving influenza vaccines in subsequent years.^3^

If replicated in other settings, these findings suggest that clinicians may wish to separate the influenza and zoster vaccines for some patients or provide specific patient education about the relative side effect profiles of the 2 vaccines, including a reminder that systemic influenza-like side effects are more likely to be caused by the zoster vaccine than the influenza vaccine. Except for a few select circumstances, the US Advisory Committee on Immunization Practices recommends simultaneous vaccination as a means of improving adherence without compromising efficacy or safety.^9^ However, these guidelines make no mention of potential future vaccine hesitancy caused by misattribution of the side effects of a more reactogenic vaccine to a less reactogenic one. In practice, vaccines are often coadministered even if the specific combination has not been studied. In our cohort, over 3000 patients received the adjuvanted zoster and adjuvanted influenza vaccines on the same day, even though coadministration of any 2 adjuvanted vaccines has not been well studied.^11^ The potential benefits of separately administering vaccines must be balanced against the known challenges in assuring adequate adherence when patients must return for vaccines on 2 separate visits. Proper counseling for those who receive both the flu and zoster vaccines could be critical for mitigating vaccine hesitancy in subsequent years.

In subgroup analyses, our results were consistent across demographic and clinical subgroups. For example, even though influenza vaccine uptake was higher among patients aged 70 years or older compared with younger patients, the association between concurrent influenza and zoster vaccines and lower subsequent vaccine uptake did not vary by age. The lone exception was that concurrent influenza and zoster vaccination had a smaller association with subsequent influenza vaccine adherence for patients who received their index influenza vaccine at a clinician’s office rather than a pharmacy. This outcome could be because a patient’s primary care physician may spend more time discussing expected vaccine side effects and reassuring patients who experience side effects. The increasing role of community pharmacies in vaccine administration offers the promise of convenience for patients,^23^ but our findings suggest that pharmacists may need to play a greater role in educating and counseling patients about expected vaccine side effects. Alternatively, these findings could be explained by unmeasured differences in socioeconomic status or education between those who received influenza vaccines at the pharmacy vs a clinician’s office because these factors are known to be associated with influenza vaccine uptake.^18^

### Strengths and Limitations

Our study has several strengths. We analyzed a large, diverse, nationally representative cohort, which allowed us to perform rigorous adjustment for confounding with 35 baseline demographic, clinical, and health care use characteristics. We implemented an active comparator design, and the control group received the same 2 vaccines as those in the exposure group, only separated by time. We performed sensitivity analyses, analyzed subgroups, and used negative controls to assess the robustness of our findings. In contrast with subsequent influenza vaccine uptake, concurrent influenza and zoster vaccine administration was not associated with lower use of other preventive services, suggesting that the difference we observed in flu vaccine uptake was unlikely to be explained by unmeasured confounding.

Our study also has some limitations. Influenza vaccine uptake was substantially higher than that in the general population,^2^ suggesting that our cohort included patients particularly prone to receiving an annual influenza vaccine. Our study may not be generalizable to populations with lower rates of baseline influenza vaccine uptake. For example, our study focused on patients with private insurance and may not generalize to more disadvantaged and racially diverse populations, such as patients on Medicaid, for whom vaccine hesitancy may be especially important. Although most influenza vaccinations are administered at a clinician’s office or pharmacy, some patients receive influenza vaccines through their employers, community health clinics, or other sites that may not be well captured in insurance claims data. Although patients were included in our cohort only if they received at least 1 influenza vaccine in a pharmacy or office setting, we may have slightly underestimated the outcome if patients received their subsequent influenza vaccine at a different site. However, we have no reason to believe that site of subsequent influenza vaccination would differ after concurrent rather than separate influenza and zoster vaccine administration.

Additionally, our primary control group included patients who received a zoster vaccine in the 6 months before their index influenza vaccine; therefore, we may have introduced selection bias by excluding patients already hesitant about the influenza vaccine who were dissuaded from receiving a 2018-2019 influenza vaccines after experiencing zoster vaccine side effects. This factor could have led us to overestimate the difference between the 2 groups. However, the results were similar when we expanded the control group to include patients who received the zoster vaccine after the index influenza vaccine and thus would not be subjected to such selection bias.

Finally, despite our attempts to adjust for several potential confounders, there may still have been residual unmeasured confounding. For example, we were unable to adjust for socioeconomic factors, which are known to be associated with influenza vaccine uptake.^18^ However, in a quantitative bias analysis, we found that the association between concurrent vaccination and lower influenza vaccine uptake could not be fully explained by residual confounding by socioeconomic status. Additionally, the null confounder-adjusted associations between the exposure and several negative control outcomes suggest that we successfully balanced the 2 groups on factors that were associated with future use of preventive health care services.

## Conclusions

In this cohort study, patients who received concurrent influenza and zoster vaccines were less likely to receive a subsequent influenza vaccine the following year compared with those who received the 2 vaccines separately. This difference may be explained by side effects caused by the zoster vaccine that patients misattributed to the influenza vaccine. If these findings are replicated in other settings, clinicians may consider administering these 2 vaccines separately or providing additional patient counseling about expected side effects in order to maximize future influenza vaccine adherence.

## Notes

Supplement. **eFigure 1.** Location of Concurrent Flu and Zoster Vaccines **eFigure 2.** Adjusted Odds of the Primary and Negative Control Outcomes Following Concurrent vs Separate Flu and Zoster Vaccines, Among Selected Subgroups **eFigure 3.** Quantitative Bias Analysis for Unmeasured Confounding by Socioeconomic Status **eTable 1.** Independent Predictors of Receiving 2019-2020 Flu Vaccine **eTable 2.** Primary and Secondary Outcomes
