# Supplementary material for: Influenza Vaccine Uptake in the Year After Concurrent vs Separate Influenza and Zoster Immunization
Source: JAMA Netw Open. 2021 Nov 19;4(11):e2135362. doi: 10.1001/jamanetworkopen.2021.35362 (PMC8605484; doi:10.1001/jamanetworkopen.2021.35362)
Supplement: Supplement. — eFigure 1. Location of Concurrent Flu and Zoster Vaccines eFigure 2. Adjusted Odds of the Primary and Negative Control Outcomes Following Concurrent vs Separate Flu and Zoster Vaccines, Among Selected Subgroups eFigure 3. Quantitative Bias Analysis for Unmeasured Confounding by Socioeconomic Status eTable 1. Independent Predictors of Receiving 2019-2020 Flu Vaccine eTable 2. Primary and Secondary Outcomes [file jamanetwopen-e2135362-s001.pdf]

Supplementary Online Content

Rome BN, Feldman WB, Fischer MA, Desai RJ, Avorn J. Influenza vaccine uptake in the year after concurrent vs separate influenza and zoster immunization. *JAMA Netw Open*. 2021;4(11):e2135362. doi:10.1001/jamanetworkopen.2021.35362

eFigure 1. Location of Concurrent Flu and Zoster Vaccines

eFigure 2. Adjusted Odds of the Primary and Negative Control Outcomes Following Concurrent vs Separate Flu and Zoster Vaccines, Among Selected Subgroups

eFigure 3. Quantitative Bias Analysis for Unmeasured Confounding by Socioeconomic Status

eTable 1. Independent Predictors of Receiving 2019-2020 Flu Vaccine

eTable 2. Primary and Secondary Outcomes

This supplementary material has been provided by the authors to give readers additional information about their work.

© 2021 Rome BN et al. *JAMA Network Open.*

eFigure 1. Location of Concurrent Flu and Zoster Vaccines

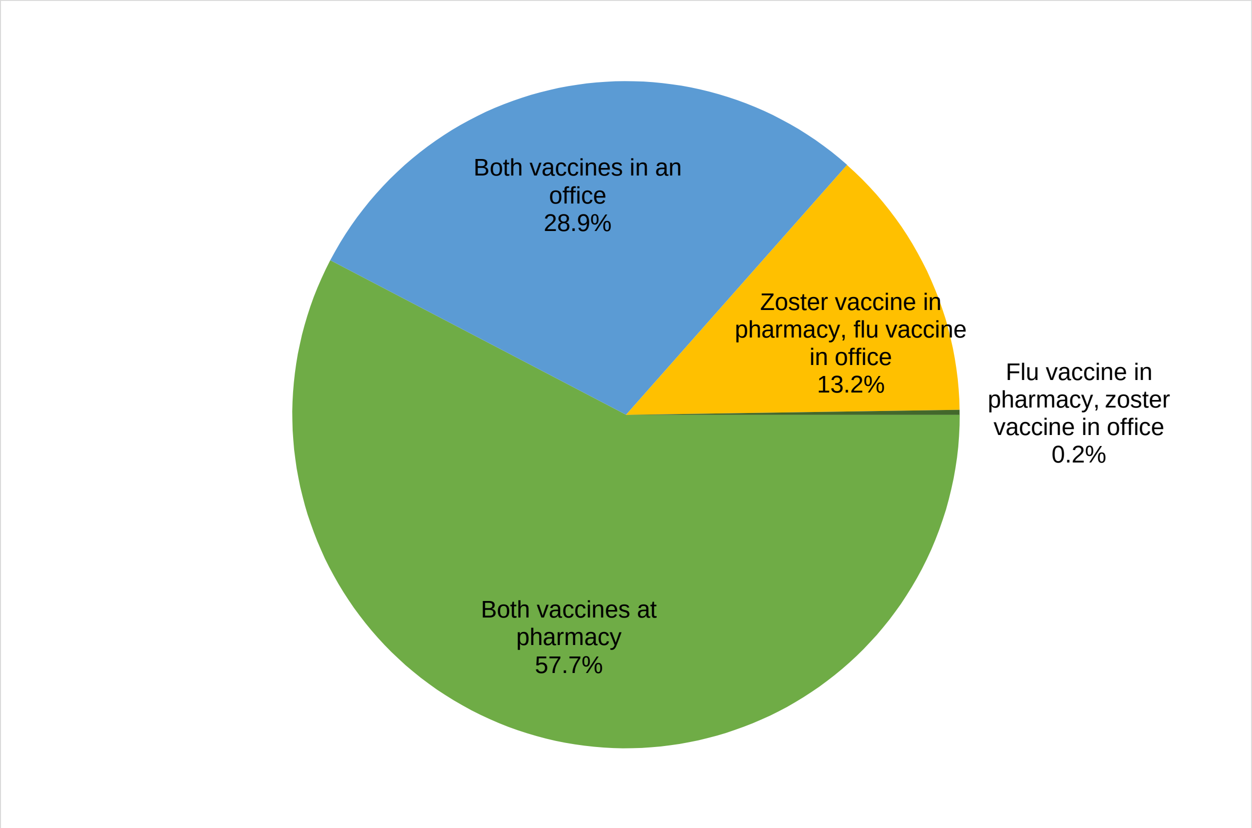

Among the 27,161 patients with same-day administration of 2018-19 flu and zoster vaccines, the figure shows where these two vaccines were . Location was determined based on whether insurance was billed through a medical claim (office) or pharmacy claim. Most patients received both vaccines at the same location. Among those who received the vaccines at different locations on the same day, most received the zoster vaccine at a pharmacy.

© 2021 Rome BN et al. *JAMA Network Open.*

eFigure 2. Adjusted Odds of the Primary and Negative Control Outcomes Following Concurrent vs Separate Flu and Zoster Vaccines, Among Selected Subgroups

Outcome [a]	Subgroup	N	Adjusted OR [b] (95% CI)	P-Value
2019-20 flu vaccine	All patients	89,237	0.74 (0.71, 0.78)	<0.001
Breast cancer screening	Women, age <75 years	32,973	0.95 (0.90, 1.01)	0.0897
Colon cancer screening	Age <75 years	56,245	1.03 (0.98, 1.09)	0.1879
Prostate cancer screening	Men, age 55-69 years	12,414	0.95 (0.87, 1.04)	0.3041
Bone mineral density testing	Women, age ≥ 65 years	43,568	0.98 (0.86, 1.12)	0.8013
Tetanus vaccination	All patients	89,237	0.99 (0.94, 1.04)	0.61
Pneumococcal vaccination	Age ≥ 65 years	74,264	1.10 (1.06, 1.15)	<0.001

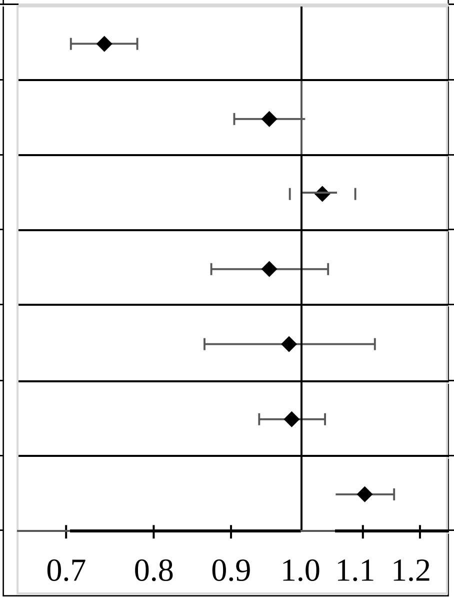

[a]The primary outcome (2019-20 flu vaccine) was measured from Aug 2019 – Mar 2020. All secondary outcomes were measured from 1 day following a patient's 2018-19 flu vaccine through Mar 2020. Each outcome was measured among the entire cohort of patients who received concurrent flu and zoster vaccines (N=27,161) and those with separate vaccines (N=62,076).
[b]The adjusted odds of experiencing the given outcome in 2019-20 following concurrent vs. separate flu and zoster vaccines in 2018-19. Each outcome model was adjusted for all the demographic, clinical, and health care utilization measures shown in Table 1. Odds ratios less than 1.0 mean that the outcome was less likely among patients with concurrent vaccines.

© 2021 Rome BN et al. *JAMA Network Open.*

eFigure 3. Quantitative Bias Analysis for Unmeasured Confounding by Socioeconomic Status

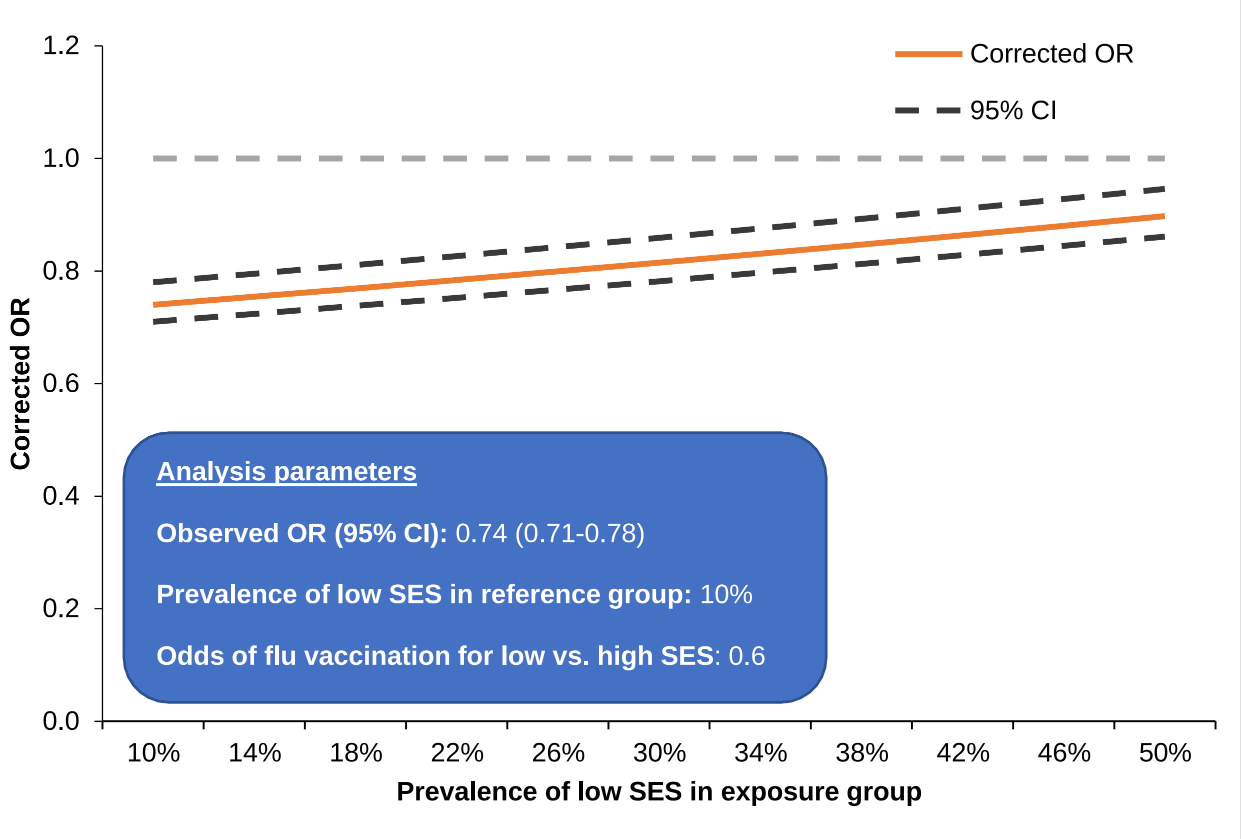

Abbreviations: CI = Confidence Interval; OR = Odds Ratio; SES = Socioeconomic status

In this analysis, we applied corrections to the odds ratio to model various scenarios of unmeasured residual confounding by socioeconomic status, using the approach of Lin et al.[1] We assumed a dichotomous SES variable (low vs. high SES), with a prevalence of low SES in the reference group (separate vaccines) of 10%. This was based on an assumption that the commercially insured population in our cohort had higher SES than the general US population, for which the poverty rate is 11.4% and 12% of adults do not have a high school diploma.[2,3] Based on published literature, we assumed that the odds ratio of flu vaccine receipt comparing low vs. high SES individuals was 0.6, based on the prevalence of flu vaccination by income level for adults aged 65 or older.[4] The figure shows the "corrected" odds ratios accounting for hypothetical residual confounding by SES, assuming a prevalence of low SES in the exposure group (concurrent vaccines) ranging from 10-50%. Even under scenarios of large differences in SES between exposure groups (up to 50% v 10%), the corrected odds ratio and the 95% confidence limits remained below 1, suggesting that the association between concurrent flu and zoster vaccination and subsequent flu vaccine uptake are unlikely to be fully explained by residual confounding by SES.

Formula:

$$\beta = \beta^{*} - \gamma\delta$$

β = unconfounded (corrected) coefficient
β^{*} = observed coefficient (confounded by SES)
γ = coefficient for association between confounder (low SES) and outcome (flu vaccine receipt)
δ = Prevalence difference in low SES between exposure group (concurrent vaccines) and reference group (separate vaccines)

© 2021 Rome BN et al. *JAMA Network Open.*

References:

1. Lin DY, Psaty BM, Kronmal RA. Assessing the sensitivity of regression results to unmeasured confounders in observational studies. *Biometrics*. 1998;54(3):948-963.

2. Ryan CL, Bauman KB. *Educational Attainment in the United States: 2015*. US Census Bureau; 2016. Accessed September 22, 2021. https://www.census.gov/library/publications/2016/demo/p20-578.html

3. Shrider EA, Kollar M, Chen F, Semega J. *Income and Poverty in the United States: 2020*. US Census Bureau; 2021. Accessed September 22, 2021. https://www.census.gov/library/publications/2021/demo/p60-273.html

4. QuickStats: Age-Adjusted Percentage of Adults Aged ≥65 Years Who Had an Influenza Vaccine in the Past 12 Months, by Poverty Status — National Health Interview Survey, United States, 1999–2001 and 2014–2016. *MMWR Morb Mortal Wkly Rep*. 2018;67. doi:10.15585/mmwr.mm6707a8

© 2021 Rome BN et al. *JAMA Network Open.*

eTable 1. Independent Predictors of Receiving 2019-2020 Flu Vaccine

**Patient characteristic**	**N**	**Percent who Received 2019-2020 Flu Vaccine**	**Adjusted OR** (95% CI)
**Timing of Flu and Zoster Vaccines**			
Separate administration	62,076	91.3	Reference
Concurrent administration	27,161	87.3	0.74 (0.71, 0.78)
**Age**			
50-59 years	7,077	83.5	Reference
60-69 years	25,084	88.8	1.00 (0.92, 1.09)
70-79 years	41,469	91.9	1.02 (0.92, 1.13)
≥80 years	15,607	90.4	0.91 (0.81, 1.02)
**Sex**			
Male	37,212	89.7	Reference
Female	52,025	90.4	0.97 (0.91, 1.04)
**Race**			
White	62,594	90.9	Reference
Black	6,358	89.6	0.81 (0.74, 0.88)
Hispanic	6,699	85.9	0.75 (0.69, 0.81)
Asian	3,576	86.8	0.73 (0.66, 0.82)
Unknown / missing	10,010	89.2	0.78 (0.73, 0.84)
**Geographic Region**			
Northeast	6,836	90.3	Reference
South	37,164	91.4	1.13 (1.03, 1.24)
Midwest	19,049	91.9	1.24 (1.12, 1.37)
West	26,188	86.8	0.70 (0.64, 0.77)
**Insurance**			
Commercial	14,388	82.6	Reference
Medicare Advantage	74,849	91.5	1.59 (1.47, 1.72)
**Received 2017-2018 Flu Vaccine**			
No	11,491	73.8	Reference
Yes	77,746	92.5	3.31 (3.14, 3.49)
**Prior Hospitalization**			
No	81,554	90.2	Reference
Yes	7,683	88.7	0.91 (0.83, 0.99)
**Prior ED Visit**			
No	70,431	90.4	Reference
Yes	18,806	89.0	0.88 (0.83, 0.94)
**Primary Care Visit**			
No	16,819	88.7	Reference
Yes	72,418	90.4	0.95 (0.89, 1.01)
**Basic metabolic panel**			
No	11,615	85.2	Reference
Yes	77,622	90.8	1.37 (1.26, 1.50)
**Complete blood count**			
No	21,124	88.3	Reference
Yes	68,113	90.6	0.99 (0.92, 1.06)

© 2021 Rome BN et al. *JAMA Network Open.*

Patient characteristic	N	Percent who Received 2019-2020 Flu Vaccine	Adjusted OR (95% CI)
**Lipid testing**			
No	20,516	87.0	Reference
Yes	68,721	91.0	1.10 (1.03, 1.18)
**Hemoglobin A1c testing**			
No	46,978	89.9	Reference
Yes	42,259	90.3	1.05 (0.99, 1.11)
**Tetanus vaccination**			
No	79,103	90.2	Reference
Yes	10,134	89.0	0.98 (0.92, 1.06)
**Pneumococcal vaccination**			
No	67,646	90.3	Reference
Yes	21,591	89.4	0.86 (0.81, 0.92)
**Colon cancer screening**			
No	79,490	90.0	Reference
Yes	9,747	90.8	1.07 (0.99, 1.15)
**Breast cancer screening**			
No	68,004	89.3	Reference
Yes	21,233	92.7	1.31 (1.23, 1.40)
**Prostate cancer screening**			
No	68,061	89.9	Reference
Yes	21,176	90.6	1.09 (1.01, 1.17)
**Bone mineral density testing**			
No	75,363	89.8	Reference
Yes	13,874	91.9	1.09 (1.01, 1.17)
**Number of Office Visits**			
0-3	19,934	88.8	Reference
4-7	27,650	90.4	0.99 (0.92, 1.05)
8-11	18,494	90.8	0.96 (0.89, 1.04)
12 or more	23,159	90.3	0.93 (0.85, 1.01)
**Number of filled prescriptions**			
0-11	14,136	86.8	Reference
12-23	19,846	90.8	1.14 (1.06, 1.23)
24-48	28,505	91.5	1.22 (1.13, 1.32)
48 or more	26,750	89.8	1.03 (0.95, 1.13)
**Diabetes**			
No	65,549	90.2	Reference
Yes	23,688	89.8	0.91 (0.86, 0.97)
**Hypertension**			
No	28,760	89.4	Reference
Yes	60,477	90.5	0.94 (0.89, 1.00)
**Cardiovascular disease**			
No	61,299	89.9	Reference
Yes	27,938	90.6	1.02 (0.96, 1.07)
**Asthma / Chronic obstructive pulmonary disease**			

© 2021 Rome BN et al. *JAMA Network Open.*

**Patient characteristic**	**N**	**Percent who Received 2019-2020 Flu Vaccine**	**Adjusted OR** (95% CI)
No	73,155	90.2	Reference
Yes	16,082	89.6	0.90 (0.85, 0.96)
**Chronic kidney disease**			
No	77,221	90.2	Reference
Yes	12,016	89.3	1.00 (0.91, 1.11)
**Liver disease**			
No	84,619	90.1	Reference
Yes	4,618	89.5	0.91 (0.85, 0.97)
**HIV**			
No	88,862	90.1	Reference
Yes	375	86.9	0.94 (0.69, 1.30)
**Chemotherapy use**			
No	78,587	90.0	Reference
Yes	10,650	91.2	1.13 (1.04, 1.23)
**Immunosuppressant**			
No	87,097	90.1	Reference
Yes	2,140	90.5	1.08 (0.93, 1.26)
**Corticosteroid use**			
No	63,322	89.9	Reference
Yes	25,915	90.7	1.05 (0.99, 1.11)
**2018-2019 Flu Vaccine Type**			
Standard dose	23,839	86.2	Reference
High dose	48,841	91.5	0.98 (0.92, 1.05)
Adjuvanted	13,711	92.8	1.05 (0.96, 1.15)
Recombinant	612	87.7	0.75 (0.58, 0.97)
Unknown	2,234	86.1	0.99 (0.86, 1.13)
**2018-2019 Flu Vaccine Location**			
Office	41,018	87.0	Reference
Pharmacy	48,219	92.8	1.46 (1.38, 1.55)
**Additional coadministered vaccine**			
No	84,221	90.4	Reference
Yes	5,016	85.0	0.82 (0.75, 0.90)
**2018-2019 Flu Vaccine Month**			
Aug 2018	6,268	90.9	Reference
Sep 2018	32,521	91.4	1.02 (0.93, 1.13)
Oct 2018	38,068	90.7	0.93 (0.84, 1.03)
Nov 2018	8,259	86.7	0.73 (0.65, 0.82)
Dec 2018	2,391	82.1	0.59 (0.51, 0.68)
Jan 2019	1,126	78.6	0.54 (0.45, 0.65)
Feb 2019	409	72.6	0.42 (0.33, 0.54)
Mar 2019	195	69.2	0.41 (0.29, 0.57)

© 2021 Rome BN et al. *JAMA Network Open.*

eTable 2. Primary and Secondary Outcomes

	**Cumulative Incidence, %** (95% CI)				
	**Concurrent Vaccines**	**Separate Vaccines**	**Risk Difference, %** (95% CI)	**Unadjusted OR** (95% CI)	**Adjusted OR** (95% CI)
**Primary Outcome (2019-2020 flu vaccine)**					
Primary analysis	87.3 (86.9, 87.7)	91.3 (91.1, 91.5)	4.0 (3.5, 4.5)	0.66 (0.63, 0.69)	0.74 (0.71, 0.78)
Sensitivity analysis	75.3 (.4.80, 75.7)	81.6 (81.3, 81.9)	6.3 (5.8, 6.9)	0.69 (0.67, 0.71)	0.84 (0.81, 0.86)
**Secondary Outcomes (Negative Controls)**					
Breast cancer screening	26.4 (25.9, 27.0)	27.4 (27.1, 27.1)	1.0 (0.4, 1.6)	0.95 (0.92, 0.98)	0.96 (0.92, 1.01)
Colon cancer screening	14.3 (13.8, 14.7)	13.2 (12.9, 13.5)	-1.1 (-1.5, -0.6)	1.09 (1.05, 1.14)	1.02 (0.97, 1.06)
Prostate cancer screening	25.8 (25.3, 26.4)	25.3 (25.0, 25.7)	-0.5 (-1.1, 0.1)	1.03 (0.99, 1.06)	0.95 (0.90, 1.00)
Bone mineral density testing	18.6 (18.2, 19.1)	20.5 (20.2, 20.8)	1.9 (1.3, 2.5)	0.89 (0.86, 0.92)	0.95 (0.91, 0.99)
Tetanus vaccination	10.3 (10.0, 10.7)	10.0 (9.8, 10.2)	-0.3 (-0.7, 0.1)	1.03 (0.99, 1.08)	0.99 (0.94, 1.04)
Pneumococcal vaccination	25.0 (24.5, 25.5)	23.6 (23.3, 23.9)	-1.4 (-2.0, -0.8)	1.12 (1.08, 1.17)	1.07 (1.03, 1.12)

The primary outcome was measured from Aug 2019 – Mar 2020. Secondary control outcomes were measured from the day after the patient's 2018-2019 influenza administration through Mar 2020. In the primary analysis and negative control analysis, only patients with continuous insurance enrollment through Mar 2020 were included. In the sensitivity analysis, patients who died or disenrolled from their insurance plan before Mar 2020 were included. All outcome models were adjusted for all demographic, clinical, and health care utilization covariates shown in figure 1. Abbreviations: CI = Confidence Interval, OR = Odds Ratio

© 2021 Rome BN et al. *JAMA Network Open.*
